# Statistical Features and Expert Knowledge for Monitoring Operating Efficiency and Conditions of Wastewater Treatment Pumps

**DOI:** 10.3390/ma12244101

**Published:** 2019-12-08

**Authors:** Chang-Ming Lin, Sheng-Fuu Lin

**Affiliations:** 1 Institute of Electrical and Control Engineering, National Chiao Tung University, No. 1001 University Road, Hsinchu 30010, Taiwan; sflin@mail.nctu.edu.tw; 2 Energy and Environment Research Laboratories, Industrial Technology Research Institute, Rm.820, Bldg.51, 8F, 195, Sec.4, Chung Hsing Rd., Chutung, Hsinchu 31040, Taiwan

**Keywords:** wastewater pump monitoring, efficiency statistical analysis, wastewater pump conditions

## Abstract

Wastewater treatment requires energy-intensive technology. The demand for industrial and residential wastewater treatment is increasing, and it has been widely accepted that low energy consumption and high operating efficiencies are essential to achieve high operating benefits in wastewater treatment plants (WWTPs). In this study, characteristic parameters of equipment operation were collected and subject to statistical analysis for trend observations in combination with expert knowledge to monitor equipment operating conditions. A methodology was developed to monitor and assess operating efficiencies of industrial equipment while not interfering with the existing operation. The proposed methodology was applied to monitor the pump efficiency in a WWTP. The results showed that the new methodology resulted in real-time acquisition of statistical operating data and enabled online detection of abnormal pump operation. The energy loss to low operating efficiencies of a malfunctioning pump was prevented, thereby allowing the pump to operate at high efficiency with an extended equipment life.

## 1. Introduction

Increasing industrial development and urbanization has led to an increased demand for sewage and household wastewater treatment, which leads to an annual increase in the demand for wastewater treatment plants (WWTPs). WWTPs must rely on long-term and intensive operation of equipment to meet both industrial and domestic needs. The main function of an inlet pumping station is to pressurize the wastewater and raise it to the corresponding WWTP for following operations. When the pump fails to operate, it affects the functions of a WWTP and causes abnormality in the operation of the biological treatment system. As a consequence, economic loss and abnormal energy consumption occur. The WWTP is a system of complex pipelines. Wastewater is collected from the sewer system or the factory pipe production line and pipelines to the wet wells of the WWTP. Wet wells located several meters below the ground are provided with multiple water pumps and their respective pipelines. The pumps use a parallel method to extract the wastewater from the wet well to the aeration tank for oxidation reaction. In the procedures of the wastewater treatment process, the wastewater treatment consumes the largest amount of power (i.e., 50% to ~80%) of the entire plant, but the sludge treatment accounts for only 15% to ~40%. Therefore, wastewater treatment is the focus of energy saving, and the main energy-consuming equipment is the pump of the WWTP. The power consumption of the pumps in the WWTP generally accounts for 10% to ~20% of the total power consumption of the wastewater treatment plant.

Since 2000, climate change has become increasingly severe, and climate has become an important factor affecting the amount of wastewater produced. Sudden rainfalls or typhoons produce a large amount of high-turbidity wastewater with many impurities in a short period of time, which increases the operating difficulty of WWTPs and prolongs reaction time. When rainfall enters the water collection well in the plant area and reaches the overflow nozzle, the water level in the sewage plant has reached the standard of designed water intake. The net head of the pump is reduced, and the lifting flow required by the pump exceeds the daily flow. The rainfall causes the actual amount of rainwater and sewage-mixed water entering the sewage treatment plant to exceed the daily water intake. During heavy rain, a large amount of sediment accumulates in the primary sedimentation tank, which increases the operating pressure of the dehydrator. The sediment also causes the sludge to rise and the wear of the dewatering equipment. At the same time, entry of a large amount of sediment also changes the nature of the sludge and the original dehydration, and therefore the medicament needs to be adjusted accordingly. Heavy rains easily cause damage to sewage treatment plant equipment, and also increase the cost of operation. 

During heavy rain or rainy season, the sewage treatment plant needs to make a series of adjustment measures to control operations. One of the important tasks is to control the flow of water by controlling the amount of water which is achieved by switching the pump or reducing the pump outlet valve. This keeps the water inflow and discharges the excess water out of the plant through the overflow pipe. Therefore, the operating conditions and efficiency of the pumps are crucial for WWTPs because failing to monitor them in time results in delayed WWTP operations and abnormal energy consumption. Moreover, severe pump malfunction is likely to cause shutdown maintenance. Monitoring of rotary equipment is primarily intended for identification of unexpected or abnormal operating conditions and behavior of equipment. To achieve this, variation of statistical operating parameters such as sound, cutting force, vibration, temperature, and energy consumption are monitored [1,2]. Such monitoring isused, for example, in the management of electricity use in a building and diagnosis of equipment malfunction. The monitoring process includes collection of operating data, data processing and analysis, and retrieval of useful information. The aim is to observe the operating trend and use it as a basis for subsequent operation management or malfunction diagnosis. Horrigan et al. [3] proposed a statistical approach to address operating efficiency gaps and improve the operating efficiency of buildings. Accordingly, residual-based exponentially weighted moving average (EWMA) charts and Shewhart charts are compared against a breakout detection algorithm to identify shifts or faults in the building performance data. An efficient detection of abnormal energy consumption can significantly reduce unnecessary energy costs. Capozzoli et al. [4] proposed a methodology that allows automatic detection of abnormal energy consumption of buildings. This automatic detection is achieved by applying statistical pattern recognition and artificial neural network ensembles to the actual energy consumption data coupled with outlier detection methods. Zhang and Ma [5] used a supervisory control and data acquisition system (SCADA) to collect data. They proposed a three-way model for wind turbine fault detection and sensor selection based on parallel factor analysis (PARAFAC), as well as a K-means classification method to monitor the collected operating data and identify the operating conditions of the wind turbines. In short, asset management based on optimal maintenance strategies contribute to the reliability and availability of equipment, thus, making the equipment more competitive. Bangalore and Patriksson [6] proposed a wind turbine maintenance management framework that utilizes operation and maintenance data from different sources to combine the benefits of age-based and condition-based maintenance scheduling. Dao et al. [7] developed a new method for condition monitoring and fault diagnosis of wind turbines based on cointegration analysis of SCADA data. In their method, the residuals obtained from the cointegration process of wind turbine data are analyzed to monitor the operating conditions and detect equipment fault. Lu, Liu, and Yan [8] presented a novel framework for the detection of structural changes in a rotating machine. This framework adopts a graph model for data modeling to represent and capture statistical dynamics in machine operations.

Pumping and blowing systems are often involved in the supply of energy for wastewater treatment. After three to five years of operation, most units in the blower system exhibit 10% to 20% lower efficiency, and poor operating efficiency leads to a substantial waste of power. Early detection of abnormal pump efficiency contributes to effective savings on power consumption and avoids subjecting pumps to fouling faults. Yang and Xu [9] proposed that fouling is the most important factor leading to efficiency degradation and that, consequently, the effect of fouling on engine efficiency should be accurately predicted. Linden [10] proposed that compressor corrosion and flow path contamination are problematic for the operation of many industrial axial compressors. Erosion or fouling of flow paths can result in reduced flow capacity and efficiency, which typically cause the flow to decrease by 3% to 5%, reaching values higher than 10% for severe abnormal conditions. Capodaglio et al. [11] proposed the stochastic system identification of sewer-flow models. Raduly et al. [12] proposed a neural network-based methodology for rapid assessment of WWTPs performance. Boyd et al. [13] proposed collecting operating data of WWTPs every five or fifteen minutes and using ARIMA to predict daily inflows. Puñal et al. [14] used an expert system (ES) to diagnose equipment operation and malfunction in WWTPs. The ES detected the operating conditions of the equipment based on online data analysis and predicted the operating trend based on the evolution of different variables and diagnosed faults. To ensure the normal operation of motors, Irfan et al. [15] developed a condition monitoring system for motor bearing fault identification based on motor stator current and voltage. Béraud et al. [16] employed daily average data in conjunction with expert knowledge to monitor the efficiency of WWTPs. Engelberth, Krawczyk, and Verl [17] presented a new model-based method for condition monitoring and diagnosis of turbo compressors, in which the characteristic operating behavior is mainly influenced by fouling.

Sewage treatment is a high-energy-consuming industry. Energy-saving effects are achieved by controlling the operation of equipment, selecting an effective aeration method, or reducing the pressure caused by frictional head loss through better pipeline design, and by monitoring the equipment operating status of the pump system and blower system to ensure that there is no abnormal energy loss. Therefore, reducing the energy consumption of a WWTP and conducting rational energy allocation is beneficial for the WWTP operation.

## 2. Materials and Methods 

The energy consumption of WWTPs is rapidly growing, and significant energy waste can occur due to the lack of proper management and diagnosis of energy efficiency [18]. The power consumption of the pump generally accounts for 10% to 20% of the total power consumption of the wastewater treatment plant, for example, it may be subjected to many different efficiency-degrading factors. For instance, blade wear, fouling, and blockage can reduce the flow (Q), which can decrease the operating efficiency of the pumps. In addition, many sewage treatment plants lack the normal operating baseline of the pump. Therefore, pumps could be working at low efficiency and high energy consumption for a long time, and a normal operating baseline can help the user to ensure whether the repaired pumps return to the normal high-performance running state. 

**Figure 1:**
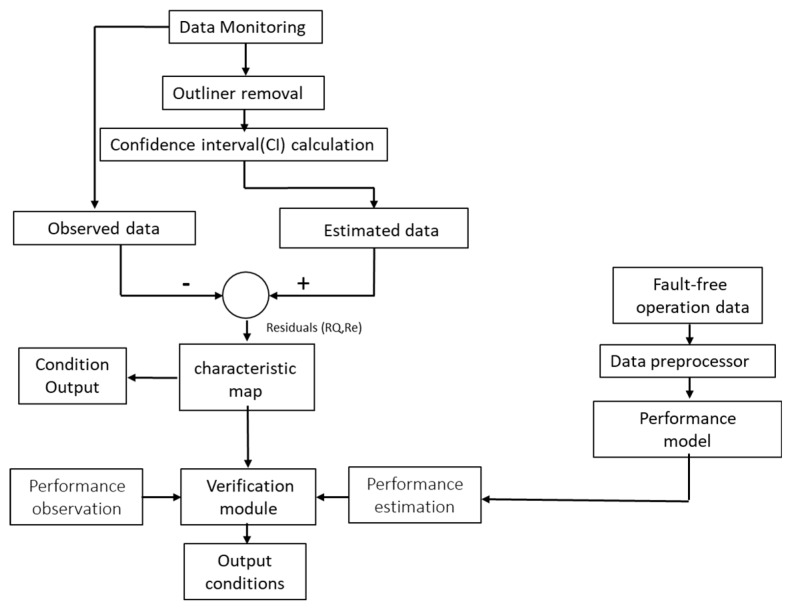
Flow chart of equipment performance monitoring.

This study combines statistical features and expert knowledge to monitor pump operating efficiency and conditions. Historical operating parameters of the equipment were collected. After the removal of outliers, parameter statistics under normal operating conditions were calculated. The obtained values were used to produce two-dimensional feature diagrams and to set warning thresholds to prevent equipment malfunction. Equipment operating conditions were monitored using the differences between the actual operating parameters and the thresholds. Moreover, to ensure the monitoring reliability, this study established a model for the energy-consumption baseline of the equipment under normal operating conditions. This baseline can ensure that there is no abnormal relationship between operating energy consumption of equipment and equipment output levels. The flow chart of the process is shown in Figure 1, and the research steps are elaborated in the following sections.

### Condition Monitoring and Diagnosis Method

In this section, a methodology suitable for WWTP pumps is proposed as follows: First, the flow (Q) and head (H) of the pump are measured because they are the main factors affecting pump efficiency (e). Secondly, two-dimensional flow-efficiency feature diagrams are produced. Thirdly, the warning thresholds of flow and efficiency for pump malfunction are determined based on statistical confidence intervals and are shown in the feature diagrams. 

**Figure 2:**
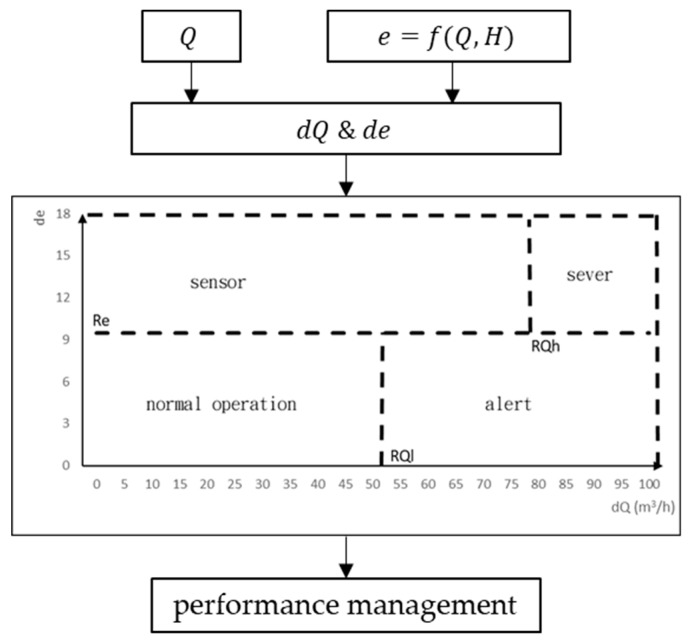
Characteristic map for performance monitoring.

The flow deficit (dQ) and the efficiency deficit (de) are calculated for real-time monitoring of the pump operating conditions. A typical two-dimensional flow-efficiency feature diagram includes three detection thresholds, namely, the high threshold of flow deficit (RQh), low threshold of flow deficit (RQl), and threshold of efficiency deficit (Re). A fault is detected when the flow or efficiency deficits of an operating condition exceeds the threshold. The thresholds are calculated using the operating parameters collected under steady operating conditions. A typical two-dimensional flow-efficiency feature diagram is illustrated in Figure 2. When a change in the pump operating conditions is detected, the extent to which the change occurs must be determined. The detection thresholds divide the two-dimensional flow-efficiency feature diagram into four regions, each corresponding to one different operating condition of the pump. For dQ < RQl and de < Re, the flow deficit and efficiency deficit are below their thresholds, which indicates that the pump is under normal conditions. There is significant correlation between pump efficiency and pump flow. However, the pipeline of a pump often contains impurities, and the flow measurement is often prone to interference by impurities. Therefore, if dQ < RQl and de > Re, the sensor is functioning abnormally. If dQ > RQl and de < Re, there is a warning of malfunction, which means that the pump flow is reduced, and the efficiency is degraded. This occurrence can be attributed to wear-ring clearance, pump operation below minimum allowable flow, impeller wear, or an improper filter device. A decrease in pump flow indicates that the pump is operating abnormally, and the more severe the pump malfunction, the higher the flow deficit, and the worse the pump efficiency. Thus, when dQ > RQh and de > Re, the pump is under severe abnormal conditions. Factors leading to flow deficit higher than RQh include wear-ring leakage, impeller corrosion, improper speed setting of variable-speed pump, reverse pump flow, and severe erosion of pump casing.

To ensure the diagnosis reliability, a regression model of pump power consumption (${\mathrm{kW}}_{\mathrm{pump}}$) versus pump flow and pump head under normal operating conditions is established. Pump power consumption is stable with respect to pump flow and is not prone to disturbance. When the power consumption (${\mathrm{kW}}_{\mathrm{esti}}$) deficit of the model-estimated pump relative to the operating pump power consumption (${\mathrm{kW}}_{\mathrm{oper}}$) exceeds the threshold of power consumption deficit ($d_{\mathrm{kW}}$), the pump is under abnormal operating conditions. Relevant symbols are elaborated in Table 1.

## 3. Results

This study combined statistics and expert knowledge to monitor equipment operating efficiencies and conditions. The proposed methodology was applied to a WWTP for the monitoring of operating efficiencies of an influent pump. The power consumption of influent pump generally accounts for 10% to 20% of the total power consumption of a WWTP [19]. Therefore, an effective monitoring of the operating conditions of the influent pump and the maintenance of good pump efficiencies can contribute to save energy and maintain normal pump operation. The studied WWTP operated 24 h a day. The start and stop of the influent pump were controlled by a level gauge. The level gauge started the influent pump when the water level was lower than a preset level, otherwise the pump was turned off.

Pumps are often subject to blockage due to the accumulation of impurities, which can also damage the pump blades, resulting in decreased pump operating efficiency. An abnormal pump condition is often identified only when there is a lack of water in the back-end treatment system. The pump would have been operating in a malfunctioning condition for a while. This would have an adverse impact on the WWTP operation, and it could lead to unnecessary power consumption and a reduction in the pump life.

**Figure 3:**
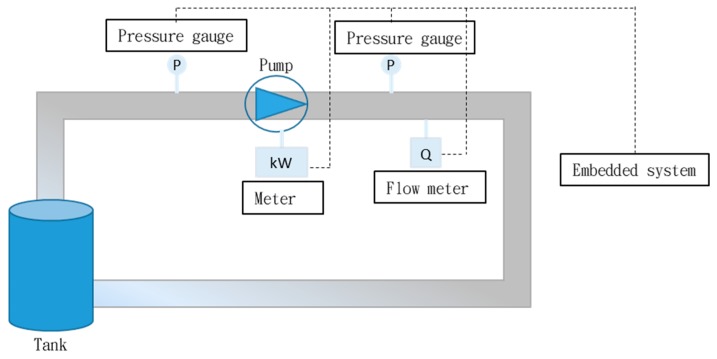
Sensor installation diagram.

**Figure 4:**
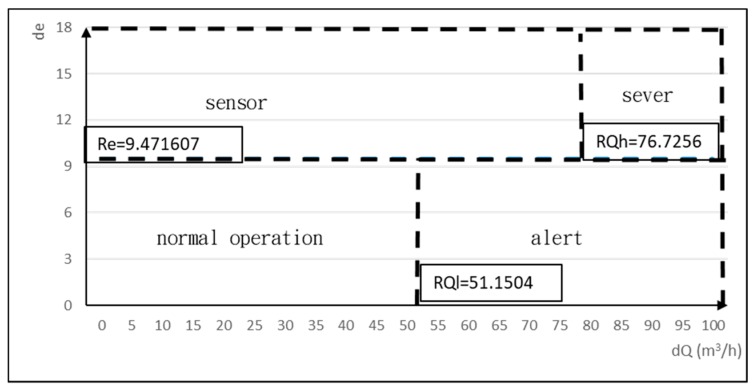
Characteristic map.

In this study, two digital pressure gauges were installed at the inlet and outlet of the influent pump, and a digital flow meter was installed on the pipeline. The operating pump flow was measured and combined with the difference between the two pressure gauges to calculate the pump head and the work (W) performed by the pump. A digital kWh meter was installed to measure the pump power consumption and calculate the pump efficiency (e). Data from the pump sensor was transmitted back to the database by an embedded device for data storage and calculation, as shown in Figure 3. Flow and pressure data of the influent pump was collected every minute in October 2018. The data collected under nonoperating conditions was removed, and the absence of outliers was confirmed. A total of 9015 normal operating datasets (collected every minute) were used to constitute a normal operating dataset of the pump. The pump work and efficiency under normal operating conditions were calculated based on the dataset analysis. Then, the daily mean of pump flow, head, power consumption, efficiency, and work, as well as the daily confidence intervals were established, as shown in Table 2. The lower limit of the confidence interval of pump flow ($C.I.L._{Q}$) and of pump efficiency ($C.I.L._{e}$) were calculated according to statistical confidence interval formula. These two limits were used as baseline parameters of normal pump operation to calculate detection thresholds (RQh, RQl, and Re). Finally, a two-dimensional flow-efficiency feature diagram was generated, as illustrated in Figure 4.

The pump consumes energy only when operating. Given that pump flow and head are the two main parameters that affect pump energy consumption, a regression model of power consumption with respect to pump flow and head was established to estimate power consumption. The regression model is expressed by Equation (1), where R^2^ is 95.99%.

(1) $${\mathrm{kW}}_{\mathrm{esti}}=0.067189{\;Q}_{\mathrm{avg}}+0.067622{\;H}_{\mathrm{avg}},$$

where $Q_{\mathrm{avg}}$ is the average daily pump flow rate, and $H_{\mathrm{avg}}$ is the daily average pump head.

**Figure 5:**
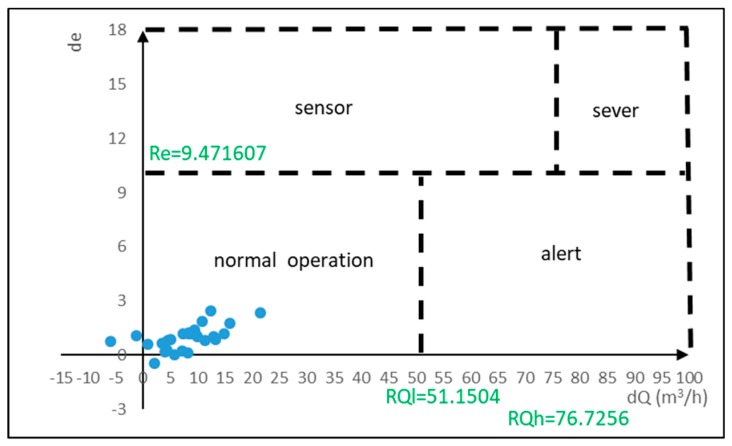
Two-dimensional flow-efficiency feature diagram in November.

**Figure 6:**
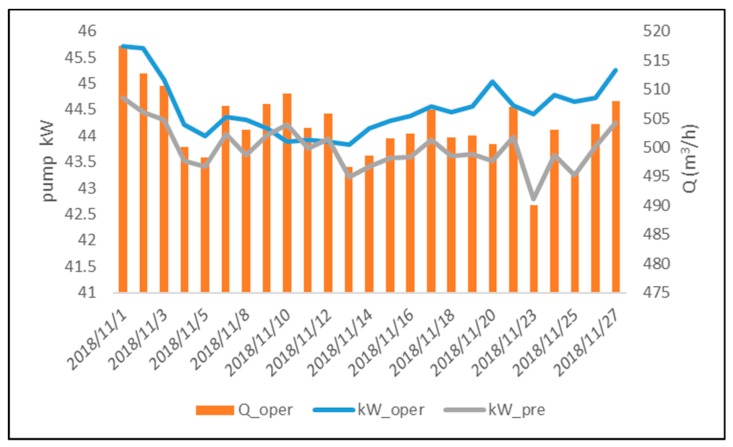
Pump power consumption versus pump flow in November.

**Figure 7:**
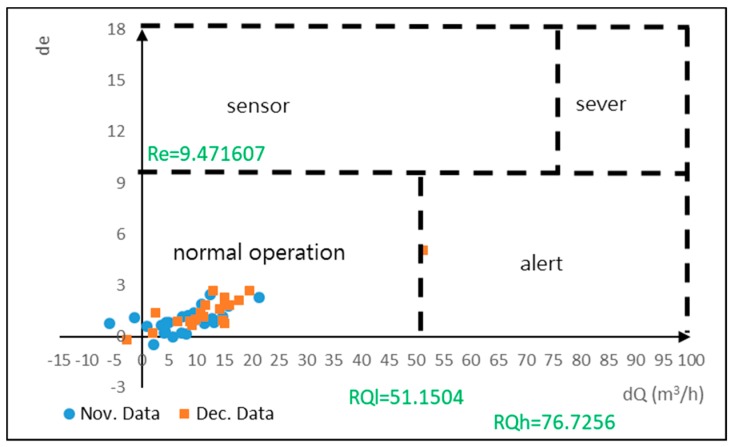
Two-dimensional flow-efficiency feature diagram of December.

**Figure 8:**
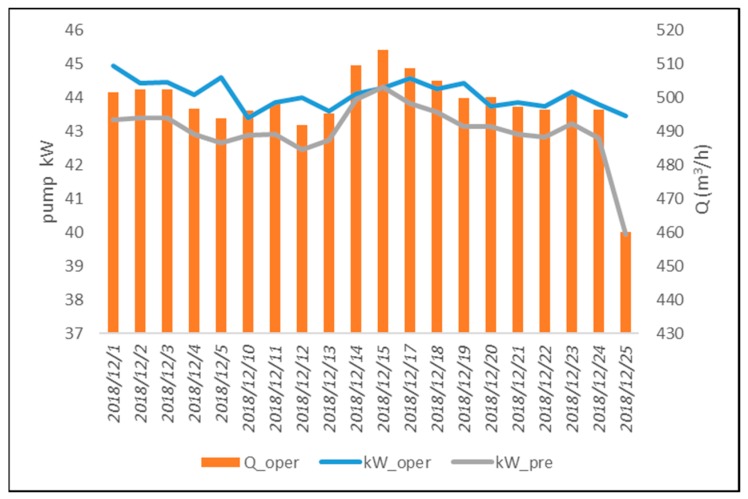
Pump power consumption versus pump flow in December.

The pump operating conditions were continuously monitored using the three thresholds established for normal pump operating conditions. The two-dimensional flow-efficiency feature diagram of the pump operation during November 2018 is shown in Figure 5. For pump operation during November 2018, the deficits of daily average flow and daily average efficiency with respect to the lower limits of the pre-established confidence intervals did not exceed the thresholds RQh, RQl, and Re. Moreover, the relative error between estimated and actual operating power consumption was less than the threshold of 7%, indicating that the pump operated normally in November. The error on pump power consumption can be seen in Figure 6. Continuous monitoring of pump operating conditions in December revealed that the pump flow deficit on December 25 was higher than the detection threshold RQl, and the efficiency deficit was lower than the detection threshold Re, indicating that the pump was operating abnormally. The regression model of pump power consumption was used to confirm the pump operating conditions. On December 25, the difference between ${\mathrm{kW}}_{\mathrm{est}}$ and ${\mathrm{kW}}_{\mathrm{oper}}$ was higher than 8%, and the actual pump flow was reduced by more than 7% as compared with previous dates under the same pump power consumption. Thus, there was a warning of malfunction conditions on December 25. The two-dimensional flow-efficiency feature diagram of pump operation in December and the estimation error of pump power consumption are shown in Figure 7 and Figure 8, respectively. 

As shown in Figure 7, the pump flow deficit that exceeded the threshold RQh occurred on 25 December 2018. The chart of pump power consumption versus pump flow (Figure 8) indicates that the pump flow on that day was far lower than the one under normal conditions, and the power consumption was within the high power-consumption range. Thus, the pump was shut down to overhaul the pumping system. It was observed that the outlet valve of the pumping system had developed a fault, which prevented it from fully opening. This fault resulted in decreased pump flow despite normal pump speed, and it caused unnecessary energy consumption.

## 4. Conclusions

Statistical features and expert knowledge were combined to monitor the operating efficiency and conditions of equipment. Operating data was collected to form a dataset of normal equipment operation and remove outliers. Statistical analysis was performed on the dataset to calculate the confidence intervals for the main operating parameters of the equipment and establish two-dimensional feature diagrams. Equipment operating conditions were monitored using the deficits of equipment operating parameters in actual equipment relative to the confidence intervals under normal operating conditions. Expert knowledge was also used in this monitoring to identify possible reasons for equipment malfunction. In addition, to ensure the reliability of equipment diagnosis, a regression model was established between equipment power consumption and statistical equipment features. The equipment operating conditions were observed, thereby allowing real-time monitoring and diagnosis of equipment operating efficiency. The proposed methodology was used to monitor the operation of an influent pump in a WWTP. It was used to identify an outlet valve fault that was degrading the pump operating efficiency. The operating efficiency of the pump dropped from 48.33 in the normal state to 45.83 in the abnormal state, and the efficiency loss reached 5.17%. Through this study, the management personnel were immediately notified to perform shutdown maintenance, thereby preventing more serious damage to the pump and unnecessary power consumption.

## Figures and Tables

**Table 1:** Main parameters.

Parameter	Description	Unit
H	$P_{\mathrm{out}}-P_{\mathrm{in}}$	kPa
$P_{\mathrm{out}}$	pump outlet pressure	kPa
$P_{\mathrm{in}}$	pump inlet pressure	kPa
W	Q × H	kW
Q	Flow	m^3^/h
e	$\left(W/{\mathrm{kW}}_{\mathrm{pump}}\right)\times 100$	
RQh	$C.I.L._{Q}-\left(C.I.L._{Q}\times 0.9\right)$	m^3^/h
RQl	$C.I.L._{Q}-\left(C.I.L._{Q}\times 0.85\right)$	m^3^/h
Re	$C.I.L._{e}-\left(C.I.L._{e}\times 0.8\right)$	
$C.I.L._{Q}$	${\bar{X}}_{Q}-1.96\sigma_{\bar{X}}$	m^3^/h
$C.I.L._{e}$	${\bar{X}}_{e}-1.96\sigma_{\bar{X}}$	
${\bar{X}}_{Q}$	mean pump flow	m^3^/h
${\bar{X}}_{e}$	mean pump efficiency	
dQ	$C.I.L._{Q}-Q$	m^3^/h
de	$C.I.L._{e}-e$	

**Table 2:** Daily averages of pump operating parameters.

	KW	W	E	Q	H
AVG	44.382	21.006	47.618	513.026	146.625
Standard error (SE)	0.084	0.037	0.126	0.741	0.220
Standard deviation (SD)	0.438	0.194	0.656	3.848	1.142
Number of samples (N)	27	27	27	27	27
Margin of error	0.173	0.065983	0.2152	0.758536	0.428848
